# Three regions of the *NIP5;1* promoter are required for expression in different cell types in *Arabidopsis thaliana* root

**DOI:** 10.1080/15592324.2021.1993654

**Published:** 2021-11-09

**Authors:** Mayuki Tanaka, Toru Fujiwara

**Affiliations:** Graduate School of Agricultural and Life Sciences, The University of Tokyo, Tokyo, Japan

**Keywords:** Promoter, boric acid, transporter, tissue-specific expression, root

## Abstract

*Arabidopsis thaliana* NIP5;1, a boric acid diffusion facilitator, is involved in the acquisition of boron (B) from soil for growth under B limitation. *AtNIP5;1* is expressed mainly in roots, where its expression is highest in the root cap and elongation zone. Here, we studied the role of the *AtNIP5;1* promoter in the expression of this gene in roots. We fused a series of *AtNIP5;1* promoter variants with deleted 5′-fragments to the *GUS* reporter gene and investigated the expression patterns by histochemical staining. We found that three regions of the *AtNIP5;1* promoter are required for specific expression in the root cap and elongation zone (−880 to −863 bp from the translation start site), distal side of the differentiation zone (−747 to −722 bp), and basal side of the differentiation zone (−661 and −621 bp). The results suggest that at least three regions of the *AtNIP5;1* promoter each confer different cell-type-specific expression.

## Introduction

A promoter is a sequence upstream of the transcription start site that often contains specific sequence motifs, including *cis*-acting elements, involved in time- and space-dependent expression, tissue- and organ-specific expression, or regulation by environmental changes. *Trans*-acting factors (transcription factors) bind to the *cis*-acting elements to regulate gene expression. Since the *cis*-acting elements are common to all cells of an organism, the specificity of gene expression is determined by the activity of transcription factors in specific organs and tissues. Information on the positions of *cis*-acting elements and on the transcription factors is invaluable in elucidating their biological functions. In crops, this regulation has the potential to determine agronomically important traits, placing this topic in the center of attention of plant biologists^1^

Since boron (B) was proven to be an essential micronutrient for plants in 1923,^2^ evidence has accumulated that it is required for normal growth not only of vascular plants, but also of diatoms, cyanobacteria, and a number of species of marine algal flagellates.^2,3^ It is also reported that excess B is toxic.^4^ In plants, B is important for maintaining cell wall structures for normal growth. Cross-linking of B with rhamnogalacturonan-II (RG-II), a complex pectic polysaccharide in the cell wall, is required for normal expansion of leaves.^5,6^ Since B is transported along the transpiration stream and is supposed to be phloem-immobile in many plants, it accumulates at the end of the transpiration stream.^7^ Thus, B deficiency and toxicity symptoms in plants are often observed in the growth of apical meristems, affecting root elongation, leaf expansion, and fertilization.^8–10^ Consequently, both B deficiency and B toxicity decrease crop yield and quality.^11^

Nodulin 26-like intrinsic proteins (NIPs) are diffusion facilitators of water and small uncharged molecules such as boric acid, silicic acid, glycerol, lactic acid urea and formamide.^12^ In *A. thaliana, NIP5;1, NIP6;1*, and *NIP7;1* are boric acid diffusion facilitators and mainly expressed in roots, nodes, and anthers, respectively.^13–15^ NIPs are well conserved among plant species. Rice *NIP3;1* and maize *NIP3;1* are orthologues of *AtNIP5;1* and are boric acid diffusion facilitators.^16,17^ *OsNIP3;1* is expressed in the roots as well as in the shoot, and is involved in B uptake from the soil and B transport in the shoots, while, zm*NIP3;1* is expressed mainly in silk, and is involved in inflorescence development under limiting B conditions.

For the mechanism of B-dependent regulation, *AtNIP5;1* is the most evident. *AtNIP5;1* is regulated at the post-transcriptional level, including via mRNA degradation and translation efficiency.^18,19^ We previously demonstrated that a minimum upstream open reading frame (ORF), AUGUAA, which contains only the start and stop codons and is present in the *AtNIP5;1* 5′ untranslated region (5′-UTR), is required for B-dependent *AtNIP5;1* expression.^19^ During translation, ribosomes are likely to stall at AUGUAA under high-B conditions, reducing translation efficiency of its main ORF and inducing mRNA degradation.^19^ The sequence of the 5ʹ-UTR is well conserved among species, especially AUGUAA and the around sequences.^19^ It is likely that B-dependent regulation via AUGUAA is conserved among species.

Histochemical analysis has shown that the *AtNIP5;1* expression pattern in roots differs among cell types. *AtNIP5;1* expression is stronger in the elongation zone than in other root zones.^13^ This finding implies that *AtNIP5;1* has different *cis*-acting elements to regulate root-cell-type–specific expression. To obtain insights into this regulation, here we conducted deletion analysis of the *AtNIP5;1* promoter and found that basal levels of *AtNIP5;1* expression in each root cell type are regulated by distinct promoter regions.

## Materials and methods

### Plant growth conditions

*A. thaliana* 4-, 5-, and 11-day-old seedlings were grown on plates with solid medium^20^ containing 1% (w/v) sucrose, 1.5% (w/v) gellan gum (Wako Pure Chemicals, Osaka, Japan), and different concentrations of boric acid (Wako Pure Chemicals). Surface-sterilized seeds were sown on the plates and incubated for 1–2 days at 4°C. The plates were then placed vertically at 22°C in a growth chamber under long-day conditions (16/8-h light/dark cycle). Twenty-one-day-old plants were grown on plates with solid medium containing 1% (w/v) sucrose, 0.1% (w/v) gellan gum and 0.3 µM boric acid. The plates were placed horizontally at 22°C in a growth chamber under long-day conditions.

### Plasmid construction and plant transformation

The *AtNIP5;1* promoter with serial deletions (Supplemental Table 1) from the 5′ end was fused to the β-glucuronidase (*GUS*) reporter gene. The P_−2492_–GUS construct carried a fragment from −2492 to +1 bp (nucleotide numbering from the translation start site) of the wild-type *AtNIP5;1* gene and the GUS reporter gene (“P_−2180_–GUS”^18^). The P_−2492∆UTR558–313_–GUS construct carried a deletion of −558 to −313 bp in the 5′-UTR of *AtNIP5;1* (“P_−2180∆UTR312_–GUS^18^). In brief, to construct P_−2492_–GUS, the region from −2492 to +1 bp was amplified by the bacterial artificial chromosome (BAC) clone F24G24 that harbors the *AtNIP5;1* locus obtained from the Arabidopsis Biological Research Center (http://abrc.osu.edu/). All primers mentioned in this subsection are shown in Supplemental Table 1. The amplified fragment was digested with *Bam*HI and *Nco*I and subcloned into pTF456 (a derivative of pBI221^21^ containing a *GUS* ORF with an *Nco*I site at its 5′-end). The resultant plasmid was named pMW1.^13^ The *Bam*HI*–Not*I fragment of pMW1 (−2492 to +1 bp fragment and the *GUS* gene) was subcloned into *Bam*HI–*Bsp*120-I–digested pTkan^+^ (provided by K. Schumacher, University of Heidelberg). To construct P_−2492∆UTR558–313_–GUS, the region from −2492 to −313 bp was amplified by PCR from pMW1.

Using the pMW1 construct as a template, we PCR-amplified the fragments of the 5′-UTR of *AtNIP5;1* starting from positions −1559, −900, −880, −863, −819, −802, −762, −747, −722, −700, −681, −661, −621, and −600 with forward primers and the M13/pUC universal primer. The amplified fragments were digested with *Bam*HI and *Not*I and subcloned into the *Bam*HI–*Bsp*120-I–digested pTkan^+^.

*A. thaliana* (L.) Heynh. ecotype Columbia (Col-0) plants were transformed using the floral-dip method.^22^ At least three independent homozygous T_3_ plants were established for each transgenic line and were used for analysis.

### Fluorometric assays of GUS expression

Transgenic lines carrying the deletion series of the *AtNIP5;1* promoter were grown on solid medium containing 0.3 or 100 µM boric acid for 5 days. To observe the *AtNIP5;1* expression pattern, the seedlings were vacuum infiltrated with GUS-staining solution (100 mM Na-PO_4_, pH 7.0, 0.1% Triton X-100, 2 mM K_3_Fe[CN]_6_, 2 mM K_4_Fe[CN]_6_, 0.5 mg/ml 5-bromo-4-chloro-3-indolyl-β-D-glucuronic acid (X-Gluc)) (Wako Pure Chemicals) for 15 min and were then kept overnight at 37°C in the dark. Pigments were cleared by treating GUS-stained seedlings consecutively with 70% and 100% ethanol. Images of the root elongation zone were acquired under a light microscope.

## Results

### Root cell type-specific AtNIP5;1 expression and its B-dependent expression are regulated by different pathways

**Figure 1.**
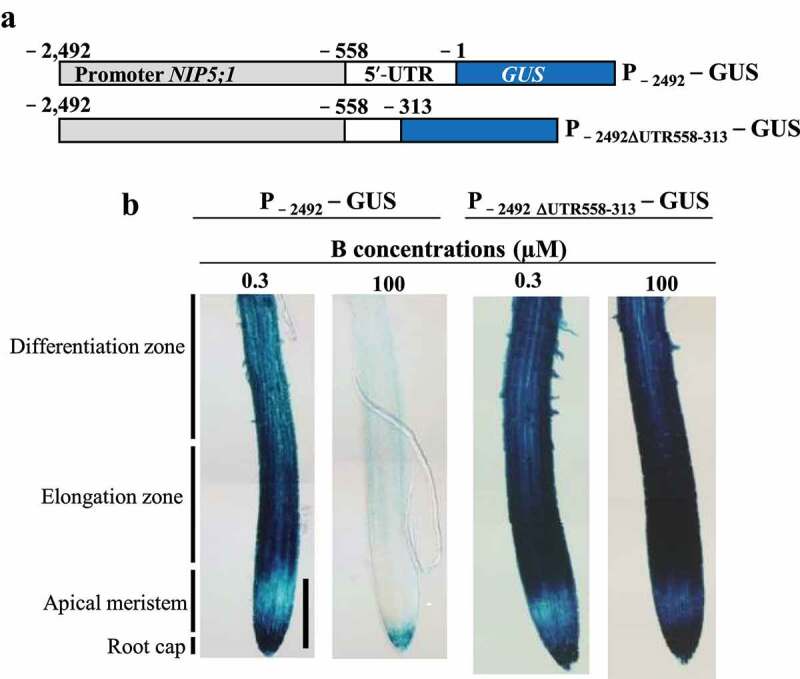
GUS expression patterns in roots of transgenic plants carrying the *AtNIP5;1* promoter with or without a deletion in the 5′-UTR in the presence of 0.3 or 100 µM B. (a) Transformation constructs carrying P_−2492_–GUS and P_−2492ΔUTR558–313_–GUS. Nucleotides are numbered from the translation start site (+1). (b) Transgenic plants were grown for 5 days in the presence of 0.3 or 100 µM B and were stained with 5-bromo-4-chloro-3-indolyl-β-d-glucuronic acid. Two or three independent transgenic lines were observed; representative images are shown. Scale bar = 200 µm.

GUS expression driven by the *AtNIP5;1* promoter with the 5′-UTR, which starts −2492 bp upstream of the main ORF (referred to as P_−2492_–GUS), is reportedly strongly induced by B deficiency in roots and is high in the elongation zone.^13^ To investigate the root-specific *GUS* expression patterns in root regions, the same transgenic plants carrying P_−2492_–GUS were grown under low (0.3 µM) or high (100 µM) B conditions (Figure 1). Under low-B, although GUS staining was observed in the whole roots, its patterns differed among the regions: it was strongest in the root elongation zone and the root cap, followed by the differentiation zone, and weak in the apical meristem region. Under high-B, the overall GUS expression was weaker, but the pattern was similar to that under low-B.

To investigate whether the *AtNIP5;1* expression pattern in roots is altered when the responsiveness to B is abolished, we examined the GUS expression pattern in transgenic plants carrying the *AtNIP5;1* promoter without a portion of 5′-UTR (referred to as P_−2492∆UTR558–313_–GUS); these plants have no B response in GUS activity assay.^18^ The GUS expression patterns in roots were similar between B conditions and similar to those in plants carrying the *AtNIP5;1* promoter with wild-type 5′-UTR under low-B (Figure 1b). This result confirms that the 5′-UTR is involved in B-dependent expression in roots and indicates that it is not involved in cell-type-specific *AtNIP5;1* expression in roots. Thus, specific promoter regions might confer distinct cell-type-specific *AtNIP5;1* expression in roots and might control basal levels of *AtNIP5;1* expression in each root region.

### Distinct promoter regions confer different cell-type-specific expression in roots

**Figure 2.**
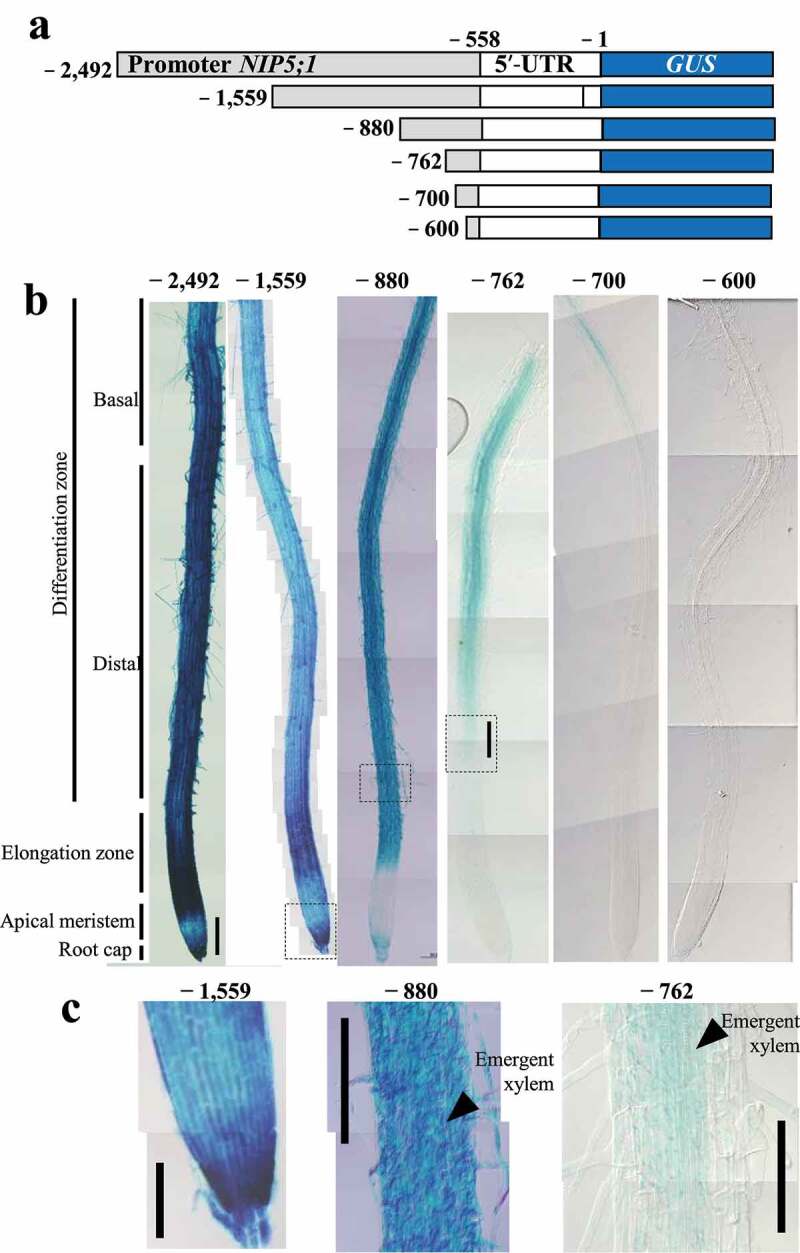
GUS expression patterns in roots of transgenic plants carrying 5′-truncated *At NIP5;1* promoter in the presence of 0.3 µM B. (a) Transformation constructs carrying *AtNIP5;1* promoter with 5′ deletions. Nucleotides are numbered from the translation start site (+1). (b, c) Transgenic plants were grown for 5 days in the presence of 0.3 µM B. (c) Enlargements of the dotted boxes in P_−880_–GUS and P_−762_–GUS in (b). The arrowheads indicate the area where the xylem is just beginning to form. Negative numbers represent construct names. Scale bars = 200 µm (b), 100 µm (c).

To investigate cell-type-specific expression in roots, we used a 5′-deletion series.^18^ The positions are −1559, −880, −762, −700, and −600, numbered from the translation start site, +1) of the *AtNIP5;1* promoter. These were fused to the *GUS* reporter gene (referred to as P_−1559_–GUS, P_−880_–GUS, P_−762_–GUS, P_−700_–GUS, and P_−600_–GUS)^18^ (Figure 2, Supplemental Figures S1 and S2). The deletion of these promoter regions except for P_−600_–GUS reportedly does not affect B response in a GUS activity assay, while GUS activity in transgenic plants carrying P_−600_–GUS is close to the detection limit.^18^

For each construct, GUS expression was observed in at least three independent lines; representative images are shown in Figure 2. The GUS staining patterns in roots differed among the lines. In those carrying P_−1559_–GUS and P_−880_–GUS, the expression patterns were almost identical to those in lines carrying P_−2492_–GUS (Figures 2b, c). No GUS expression was observed in the apical meristem in the lines carrying P_−880_–GUS, unlike in those carrying P_−2492_–GUS and P_−1559_–GUS, presumably owing to a difference in the level of GUS expression among lines. On the other hand, GUS expression in lines carrying P_−762_–GUS was undetectable in the root cap and elongation zone, but was detectable from the region where xylem emerges (Figure 2c). GUS expression in lines carrying P_−700_–GUS was observed only in the upper part of the differentiation zone (i.e., in the basal root region). No GUS expression was detectable in lines carrying P_−600_–GUS.

These trends were also observed in 11-day and 21-day-old plants (Supplemental Figure S2). Interestingly, it is likely that GUS expression in lines carrying P_−700_–GUS was observed when the lateral roots started to emerge (Supplemental Figure S2A). It suggests that the difference between the upper and lower part of the differentiation zone is due to the difference in the area where the lateral roots can emerge or not. In addition, as is the case of the main root, the expression pattern in the lateral root was also regulated by the promoter region (Supplemental Figures S2C and S2D). These observations suggest that the *AtNIP5;1* promoter region contains different elements for root cell-type-specific *AtNIP5;1* expression, including expression in the root cap elongation zone, and the distal and basal regions of the differentiation zone.

### Identification of AtNIP5;1 promoter regions required for cell-type-specific expression in roots

**Figure 3.**
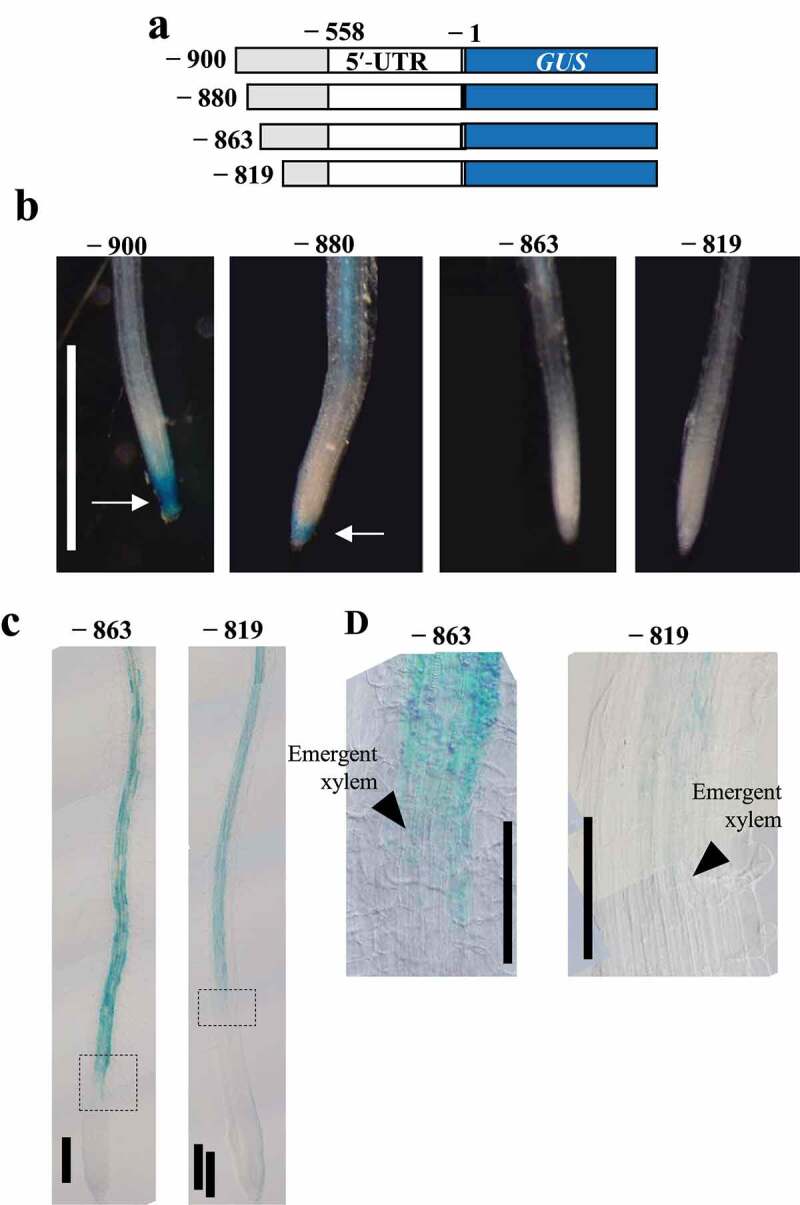
GUS expression patterns in the root cap and elongation zone of transgenic plants carrying truncated *AtNIP5;1* promoter with deletions in the region from −900 to −819 bp. (a) Transformation constructs carrying *AtNIP5;1* promoter with 5′ deletions. Nucleotides are numbered from the translation start site (+1). (b, c) Transgenic plants were grown for 5 days in the presence of 0.3 µM B. Arrows indicate stained root caps. (d) Enlargements of the dotted boxes of P_−863_–GUS and P_−819_–GUS in C. The arrowheads indicate the area where the xylem is just beginning to form. Negative numbers represent construct names. Scale bars = 1 cm (a), 200 µm (b), 100 µm (c).

To investigate *AtNIP5;1* expression specific to the root cap and elongation zone, we made transgenic plants with promoters starting before or after position −880, namely, at positions −900, −863, or −819 of the *AtNIP5;1* promoter. These regions were fused with *GUS* (referred to as P_−900_–GUS, P_−863_–GUS, and P_−819_–GUS; Figure 3a). We basically made constructs with deletions of roughly 20 bp interval.

GUS expression was observed in the root cap in lines carrying P_−900_–GUS and P_−880_–GUS, but not in those carrying P_−863_–GUS or P_−819_–GUS (Figure 3b). In addition, in lines carrying P_−880_–GUS the expression was observed in the elongation zone (Figure 2b), whereas in lines carrying P_−863_–GUS and P_−819_–GUS it was observed in and above the region where the xylem appears (Figures 3c,d). These data indicate that the sequence from −880 to −863 is necessary for *AtNIP5;1* expression specific to the root cap and elongation zone.

**Figure 4.**
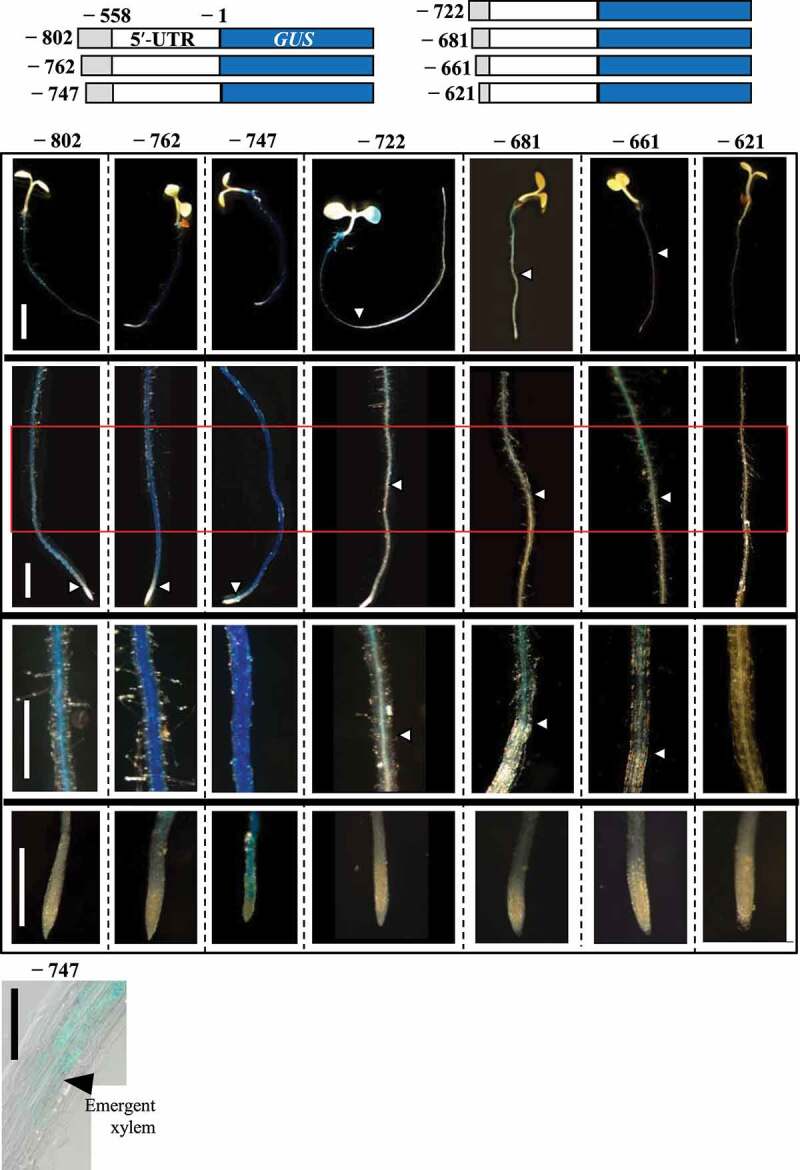
GUS expression patterns in roots of transgenic plants carrying truncated *AtNIP5;1* promoter with deletions in the region from – 802 to – 621 bp. (a) Transformation constructs carrying *AtNIP5;1* promoter with 5′ deletions. Nucleotides are numbered from the translation start site (+1). Plants were grown for 4 to 5 days in the presence of 0.3 µM B. (b) Whole plants. (c) Roots. (d) Enlargements of the red boxes (differentiation zones) in C. (e) Root caps. (f) Enlargement of the region where xylem emerges in P_−747_–GUS. The black arrowhead indicates the area where the xylem is just beginning to form. Arrowheads in (b–d) indicate the positions above which GUS expression was detected. Negative numbers represent construct names. Scale bars = 10 mm (b), 1 mm (c), 0.5 mm (d, e), 100 µm (f).

Next, to examine *AtNIP5;1* expression specific to the differentiation zone, we made transgenic plants with promoters starting before or after position −762 and −700 with roughly 20 bp interval, namely at positions −802, −747, −722, −681, −661, or −621 of the *AtNIP5;1* promoter. These regions were fused to *GUS* (referred to as P_−802_–GUS, P_−747_–GUS, P_−722_–GUS, P_−681_–GUS, P_−661_–GUS, and P_−621_–GUS; Figure 4).

GUS staining in lines carrying constructs from P_−802_–GUS to P_−747_–GUS was observed throughout the differentiation zone, i.e., from where the xylem appears to the upper part (Figure 4f). On the other hand, GUS expression in lines carrying P_−722_–GUS to P_−661_–GUS was observed only in the basal part of the differentiation zone (Figure 4). No GUS expression was detected in lines carrying P_−621_–GUS. These results show that sequences from −747 to −722 bp and from −680 to −661 bp are necessary for specific *AtNIP5;1* expression in the distal and basal parts, respectively, of the differentiation zone.

## Discussion

**Figure 5.**
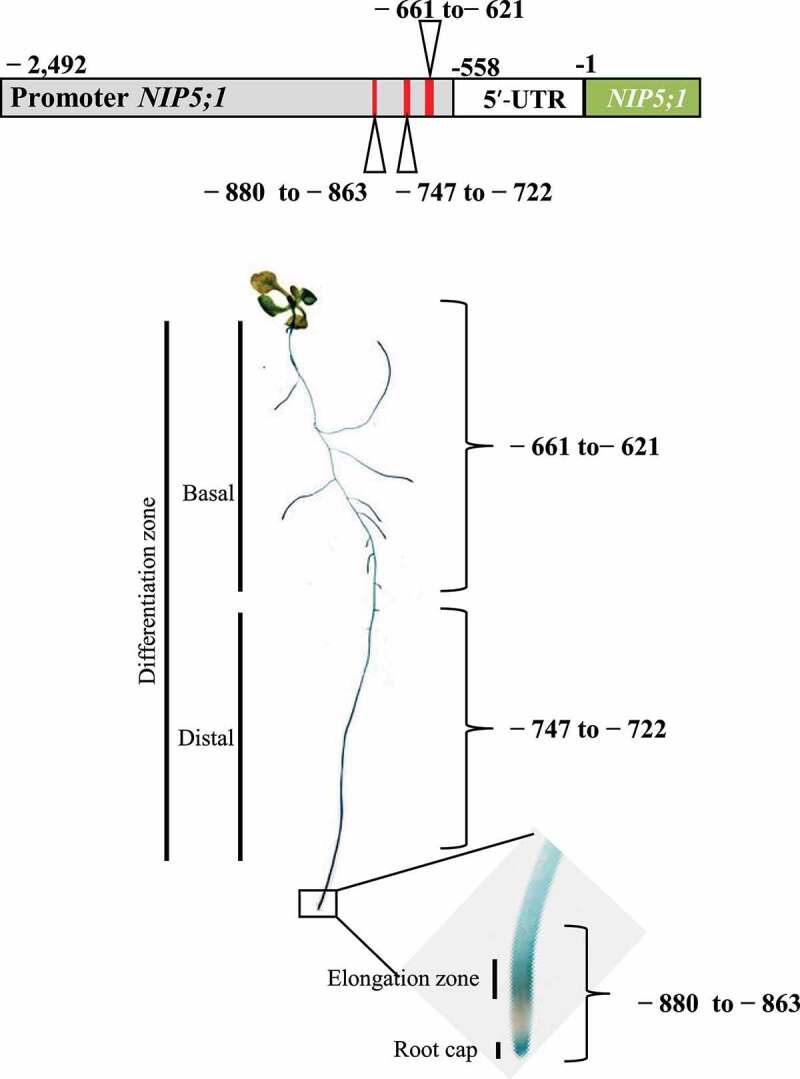
Positions of the regions that confer root-cell-type–specific expression. The −880 to −863 bp region is required for expression in the root cap and elongation zone, the −747 to −722 bp region for expression in the distal part of the differentiation zone, and the −661 to −621 bp region for expression in the basal part of the differentiation zone.

Here we show that three distinct promoter regions are required for cell-type-specific *AtNIP5;1* expression in roots namely in the root cap and elongation zone (−880 to −863 bp from translation start site), distal part of the differentiation zone (−747 to −722 bp), and basal part of the differentiation zone (−661 to −621 bp) (Figure 5). B-unresponsive transgenic plants carrying a partial deletion of the 5′-UTR had higher GUS activity in the root cap and elongation zone than in the other root zones under both B conditions (Figure 1). Thus, whereas B-dependent *AtNIP5;1* expression is regulated by ribosome stalling at AUGUAA at the 5′-UTR,^19^ the basal levels of *AtIP5;1* expression in different root cell types are regulated by the promoter.

For regulatory elements in the *AtNIP5;1* promoter, we searched the Plant Promoter Database (Ppbd)^23^ and identified three such elements at −844 to −837 bp, −768 to −760 bp, and −646 to−639 bp. These positions are very close or almost identical to those we identified in this study. The −844 to −837 bp sequence is a W-box motif recognized by WRKY transcription factors in response to salicylic acid.^24^ The −767 to −761 bp sequence is the site YAACKG or CNGTTR recognized by MYB transcription factors in response to dehydration.^25,26^ The W-box motif and MYB recognition site are slightly out of alignment with the regions we identified, and their functions contribute to the stress response. Thus, they are unlikely to be involved in root-cell-type-specific expression, and other transcription factors might be involved. In Ppbd, the −646 to −639 bp sequence is listed as a regulatory element whose function is unknown. This sequence lies within our identified region and may be essential for specific expression in the basal part of the differentiation zone.

We compared the promoter regions between *AtNIP5;1* and its rice ortholog *OsNIP3;1*, but the similarity was low and the root-specific sequences found in *AtNIP5;1* were not detected in *OsNIP3;1*. The latter is also a boric acid facilitator and expressed in both shoots and roots.^16^ In *A. thaliana, AtNIP6;1*, a paralog of *AtNIP5;1*, is expressed mainly in shoots, especially in the nodal region.^14^ The expression pattern of *OsNIP3;1* corresponds to the combined patterns of *AtNIP5;1* and *AtNIP6;1*. Rice, sorghum, and maize have only AtNIP5;1 orthologs, whereas soybean, citrus, grape, and poplar have orthologs of both AtNIP5;1 and AtNIP6;1.^27^ It seems likely that *NIP5;1* and *NIP6;1* diversified during evolution, and their promoter regions were altered and gained tissue-specific expression. We hypothesize that plants have evolved to adjust to a variety of environments by the species-specific evolution of *NIP* genes. It seems likely that root-specific expression of *AtNIP5;1* became regulated differently in the three root portions, achieving further functional differentiation to fulfill the demand for B in each portion of the root.

The physiological roles of AtNIP5;1 may differ in different portions of the root. One possible role for AtNIP5;1 is B transport for RG-II–B dimer formation for cell expansion, and the other one is B transport to shoots. In radish root caps, RG-II is present mainly on the inner surface of the primary cell wall, very close to the plasma membrane.^28^ *At*BOR2, an efflux-type B transporter, is involved in RG-II–B dimer formation and is expressed in the root cap and elongation zone.^29^ According to the coexpression database Atted II,^30^ *AtNIP5;1* is coexpressed with *AtBOR2*. Given that B is relatively immobile in the phloem,^31^ B required for cell expansion must be transported from the root cap or elongation zone; thus, region-specific *AtNIP5;1* expression would be necessary to fulfill the B requirement for RG-II–B dimer formation.

*AtNIP 5;1* expressed in the differentiation zone may be involved in B transport to the xylem and shoots. We found that specific *AtNIP5;1* expression in the basal and distal parts of the root differentiation zone was regulated by different promoter regions. The basal part of the roots is attached to the leaves and is where the lateral roots are emerged. The presence or absence of B transported from the lateral roots may be responsible for the differential expression of root differentiation regions. Since the requirements for B differ among root regions, the possible need to control the basal level of *AtNIP5;1* expression in each root tissue may have led to the development of regulation by different promoter regions.

In conclusion, our analysis indicates that root-specific expression of *A. thaliana AtNIP5;1* is governed by three distinct root-cell-type-specific elements, which are responsible for expression in the root cap and elongation zone, in the distal part of the differentiation zone, and in its basal part.

## Supplementary Material

Supplemental Material
