# Supplementary material for: Three regions of the NIP5;1 promoter are required for expression in different cell types in Arabidopsis thaliana root
Source: Plant Signal Behav. 2021 Nov 9;16(12):1993654. doi: 10.1080/15592324.2021.1993654 (PMC9208793; doi:10.1080/15592324.2021.1993654)
Supplement: Supplemental Material [file KPSB_A_1993654_SM8651.pdf]

Supplemental Table1. List of Primers

Experiment	No.	genes or construction names		sequence
5′ -UTR wild-typ	1	P_{-2492}–GUS	forward (BamHI)	5′ -GGTGGATCCGAAAGCAAGCATTCCCTG -3′
	2	P_{-2492}–GUS	reverse (Nco I)	5′ -GAGCCATGGTTCCAACGTTTTTTTTTTTG-3′
5′ -UTR deletion	3	$P_{-2492\triangle UTR558-313}$–GUS	forward (BamHI)	5′ -GGTGGATCCGAAAGCAAGCATTCCCTG -3′
	4	$P_{-2492\triangle UTR558-313}$–GUS	reverse (Nco I)	5′ -GAGCCATGGGCTTTGAAAGATTTTTATAAAGC-3′
promoter deletion	5	P_{-1559}–GUS	forward (BamHI)	5′ -TTAGGATCCTGCGGACCTAAGTTATGTTG-3′
	6	P_{-900}–GUS	forward (BamHI)	5′ -TTAGGATCCATCAAACGCTCTTCCCTAGA-3′
	7	P_{-880}–GUS	forward (BamHI)	5′ -GTCGGATCCCTTAAGAGAAATTTTATA-3′
	8	P_{-863}–GUS	forward (BamHI)	5′ -GACGGATCCATTCAATTATTGGAGGGT-3′
	9	P_{-819}–GUS	forward (BamHI)	5′ -AATGGATCCTCACCGCGCATGTGCAATT-3′
	10	P_{-802}–GUS	forward (BamHI)	5′ -GTCGGATCCTTCCTAACCGTCACACACGGA-3′
	11	P_{-762}–GUS	forward (BamHI)	5′ -GTCGGATCCCGGTAACGAATCTGCCT-3′
	12	P_{-747}–GUS	forward (BamHI)	5′ -AATGGATCCCTGTGACAAAAGCACCGGTG-3′
	13	P_{-722}–GUS	forward (BamHI)	5′ -GTCGGATCCAGCAGTTCAACAAAATCTCA-3′
	14	P_{-700}–GUS	forward (BamHI)	5′ -ATCGGATCCAAGAGAGTCAGTAGATGA-3′
	15	P_{-681}–GUS	forward (BamHI)	5′ -GTCGGGATCCTGAAGCTGTTATATAAAAGC-3′
	16	P_{-661}–GUS	forward (BamHI)	5′ -GTCGGATCCAATAAGATATTGGAGTTGAC-3′
	17	P_{-621}–GUS	forward (BamHI)	5′ -GTCGGATCCTTTTTTGGGTTCAATGCATA-3′
	18	P_{-600}–GUS	forward (BamHI)	5′ -GTCGGATCCTAATCTCTTTTCTATATA-3′
	19	M13/pUC universal primer	reverse	5′ -TGGCCGTCGTTTTACACC-3′
